# Calcium-Induced Regulation of *Sanghuangporus baumii* Growth and the Biosynthesis of Its Triterpenoids

**DOI:** 10.3390/jof11030238

**Published:** 2025-03-20

**Authors:** Zengcai Liu, Ying Yu, Shiyuan Wang, Li Zou

**Affiliations:** College of Forestry, Northeast Forestry University, Harbin 150040, China; 1758458181@nefu.edu.cn (Z.L.); yuying00@nefu.edu.cn (Y.Y.); 2022110328@nefu.edu.cn (S.W.)

**Keywords:** calcium, *Sanghuangporus baumii*, triterpenoid, *AACT* gene, heterologous biosynthesis, *Saccharomyces cerevisiae*

## Abstract

*Sanghuangporus baumii*, a fungus used in traditional Chinese medicine, produces important pharmacological compounds such as triterpenoids, but at levels significantly lower than those required for medical use. This study investigated the effects of various concentrations of Ca^2+^ on *S. baumii* mycelial growth and the heterologous biosynthesis of *S. baumii* triterpenoids. Under induction by 10 mM Ca^2+^, the growth rate (0.39 cm/d) and biomass (4.48 g/L) of *S. baumii* mycelia were 1.03% and 10.05% higher than those in the 0 mM Ca^2+^-treatment group, respectively. In contrast, 200 mM Ca^2+^ significantly inhibited the growth rate and biomass of the mycelia. Notably, the total triterpenoid content reached its peak (17.71 mg/g) in the 200 mM Ca^2+^-treatment group, with a significant increase in the Ca^2+^ content (3869.97 µg/g) in the mycelia. Subsequently, the differential metabolic pathways and related genes between the *S. baumii* groups were examined using transcriptomic analysis. The results indicated that the increase in the growth rate and biomass of *S. baumii* mycelia was primarily due to elevated soluble sugar content, whereas the growth inhibition was associated with the toxic effects of H_2_O_2_. The observed differences in triterpenoid content were mainly attributed to the activation of the terpenoid backbone biosynthesis pathway and the *AACT* gene. Finally, the *AACT* gene was cloned and transformed into yeast cells, thus creating strain Sc-AA1. Upon treatment at the optimal Ca^2+^ concentration, the squalene content of strain Sc-AA1 reached 0.78 mg/g, 2.89-fold higher than that in the control group. These findings are significant for the heterologous biosynthesis of triterpenoids from *S. baumii*. Our study demonstrates the feasibility of producing triterpenoids in *Saccharomyces cerevisiae* and provides a foundation for future optimization toward achieving industrially relevant yields.

## 1. Introduction

*Sanghuangporus baumii* (Pilát) L.W. Zhou and Y.C. Dai, a fungus named for its parasitism on *Syringa reticulata* [1], was first documented in China’s classical pharmacological text “*The Divine Farmer’s Materia Medica*” and is considered a rare fungus in traditional Chinese medicine [2]. *S. baumii* exerts various beneficial effects on human health, including antitumor [3] and antioxidant effects [4], as well as life prolongation [5]. Currently, *S. baumii* is internationally recognized as one of the most effective large medicinal fungi for its anticancer properties, with triterpenoids identified as the key active components [6,7]. However, the natural yield of triterpenoids is low, which, compounded by the scarcity of wild resources, significantly limits their medicinal applications [8]. Thus, enhancing the yield of *S. baumii* triterpenoids has become an urgent challenge for researchers.

Chemical synthesis of simple terpenoids, as a direct method to produce these compounds, has seen relatively slow progress [9,10]. Because triterpenoids are complex compounds, their synthesis via chemical methods is inherently slow. In contrast, molecular biology methods present certain advantages. It has been established that triterpenoids are synthesized through the mevalonate pathway. However, this pathway involves multiple genes and necessitates the identification of key genes under varying operational conditions [11]. The exogenous addition of metal ions has emerged as an effective strategy to induce triterpenoid accumulation and facilitate the identification of key genes [12]. Xu et al. [13] demonstrated that Ca^2+^ induction increased the content of the triterpenoid GAs by 3.7-fold, likely by enhancing the expression of the *sqs* gene in *Ganoderma lucidum*. Additionally, they reported that Na^+^ regulation resulted in a 2.8-fold increase in total triterpenoid content, which was positively correlated with the transcript level of the *hmgr* gene [14].

Once the key genes involved in *S. baumii* triterpenoid biosynthesis are identified, heterologous overexpression of key genes in yeast would facilitate the safe production of triterpenoids in a short timeframe through high-density fermentation [15]. Miettinen et al. [16] co-expressed the *GgGAS* and *CYP*450 genes in engineered yeast cells and demonstrated that *CYP7*16*A*140 oxidizes the C-28 position of β-coumarin to produce the triterpenoid (oleanolic acid). Similarly, Dai et al. [17] introduced the oleic acid synthase, truncated 3-hydroxy-3-methylglutaryl-CoA reductase, *CYP*450, and related genes into yeast and reported a yield of 21.4 mg/L of oleanolic acid. These findings demonstrate the feasibility of reconstructing the *S. baumii* triterpenoid biosynthesis pathway in yeast for the production of triterpenoids or their direct precursors, aligning with future trends in the development of biosynthetically active compounds [15,18].

Research indicates that Ca^2+^, a ubiquitous second messenger in eukaryotic cells, transmits signals by increasing intracellular Ca^2+^ concentrations. These signals are conveyed to downstream effector proteins, which regulate various cellular processes, including gene transcription, enzyme activation, protein transport, and other critical reactions. To date, there have been no reports on the effects of Ca^2+^ induction on *S. baumii* mycelia or the heterologous biosynthesis of *S. baumii* triterpenoids. In light of this, this study first investigated the effects of various concentrations of Ca^2+^ on *S. baumii* mycelial growth and triterpenoid content. Next, transcriptome sequencing was used to analyze the potential changes in the triterpenoid pathway induced by Ca^2+^ and to identify the key related genes. Finally, the heterologous biosynthesis of *S. baumii* triterpenoids was achieved by transferring key genes into yeast. The present findings are important for the large-scale production of triterpenoids derived from *S. baumii*.

## 2. Materials and Methods

### 2.1. Strain and Vector

*S. baumii* strain DL101 was preserved in the protection laboratory of Northeast Forestry University. Internal transcribed spacer identification results have been submitted to NCBI GenBank (no. KP974834). *Escherichia coli* DH5α and yeast (*Saccharomyces cerevisiae* INVSc1) competent cells were purchased from WEIDI Biotechnology Co., Ltd. (Shanghai, China). The yeast expression vector pYES2-NTC was purchased from Invitrogen (Carlsbad, CA, USA).

### 2.2. Treatment of S. baumii Mycelia with Ca^2+^

To investigate the effect of Ca^2+^ on the morphology and growth rate of *S. baumii*, sterile CaCl_2_ solutions of various concentrations were prepared in accordance with previously established methods [13]. The final concentrations of Ca^2+^ in potato dextrose agar (PDA) were 10, 20, 100, 200, and 400 mM. Subsequently, fungal cakes of *S. baumii* (1 cm diameter) were placed onto the PDA medium containing the various concentrations of Ca^2+^ (Ca10, Ca20, Ca100, Ca200, and Ca400, respectively); the Ca0 group cultures were treated with equivalent volumes of sterile water. The cultures were incubated at 25 °C in the dark, and their growth rates were determined.

To examine the effect of Ca^2+^ on triterpenoid production in *S. baumii* mycelia, the mycelial liquid seed was uniformly homogenized and inoculated into 250 mL of potato dextrose (PD) medium (inoculum 4% *v*/*v*). Following inoculation, the triangular flasks containing the cultures were incubated for 8 days at 25 °C with shaking at 180 rpm. Then, Ca^2+^ was added to the PD medium to achieve final concentrations of 0, 10, 20, 100, 200, and 400 mM, and cultivation continued for an additional 2 days. The harvested *S. baumii* mycelia were subsequently used to determine the biomass, triterpenoid content, Ca^2+^ content, and other related indicators, and they were subjected to transcriptome analysis.

### 2.3. Quantification of Growth Rate, Biomass, Triterpenoid Content, and Ca^2+^ Content

After culturing *S. baumii* mycelia on PDA medium for 9 days, the growth rate was calculated by averaging the measurements of vertical and horizontal colony diameters. To determine the biomass, the *S. baumii* mycelia harvested from the PD medium were oven-dried at 45 °C until a constant weight was achieved and then weighed. The total triterpenoid content was measured following a previously described method [19]. This method is based on oxidizing the phenolic hydroxyl group of triterpenoids to a carboxyl group. Subsequently, the double bond undergoes displacement and bimolecular condensation, resulting in a conjugated system. Finally, a carbocation salt forms and develops color under acidic conditions. In brief, 0.1 g of dry cell weight of mycelia was used to extract total triterpenoids. The absorbance of the reaction solution was measured in a spectrophotometer at 551 nm, and the total triterpenoid content was measured. Ca^2+^-regulated triterpenoid biosynthesis was achieved through intracellular calcium signaling [13]. Therefore, the Ca^2+^ content of *S. baumii* mycelia was quantified using inductively coupled plasma mass spectrometry (iCAP TQ, Thermo Fisher Scientific, Waltham, MA, USA) following standard protocols [20].

### 2.4. Transcriptome Sequencing and qRT-PCR Validation

Total RNA was extracted from *S. baumii* samples that exhibited significant differences in growth and triterpenoid content following Ca^2+^ treatment; RNA extraction was performed using an RNAiso Plus Kit (Takara, Dalian, China) according to the manufacturer’s instructions. RNA quality was assessed, and quality-checked RNA was sent to PANOMIX Biomedical Tech Co., Ltd. (Suzhou, China) for transcriptome sequencing on the Illumina HiSeq 2500 platform. Subsequently, the raw reads were filtered to obtain high-quality reads, which were then assembled de novo to generate transcript sequences. The longest transcript from each cluster was designated as a unigene. Next, these unigenes were annotated, and differential expression analysis was performed, with gene expression levels estimated as fragments per kilobase per transcript per million mapped reads (FPKM). Principal component analysis (PCA) and Kyoto Encyclopedia of Genes and Genomes (KEGG) analysis were performed. Genes with |log_2_ Fold-change| > 1 and *p* < 0.05 were identified as differentially expressed genes (DEGs). Quantitative reverse transcription (qRT)-PCR was performed to validate the accuracy of the DEG identification through transcriptome analysis [21].

### 2.5. Quantification of Soluble Sugar and H_2_O_2_ Content

The changes in soluble sugar and H_2_O_2_ content are key indicators of abiotic stress. To analyze the metabolic differences in *S. baumii* mycelia under Ca^2+^ treatment, the soluble sugar content was determined. In brief, 0.1 g of mycelial sample was homogenized in 1 mL of distilled water, and the homogenate was incubated at 95 °C in a water bath for 10 min. Subsequently, the homogenate was centrifuged at 8000× *g* for 10 min. Next, 0.1 mL of the supernatant was mixed with 0.9 mL of distilled water, and the soluble sugar content was determined using a KT-2-Y kit (Suzhou Comin, Suzhou, China) according to the manufacturer’s instructions. To determine H_2_O_2_ content, 0.1 g of mycelial sample was homogenized in 1 mL of acetone in an ice bath. The homogenate was centrifuged at 8000× *g* for 10 min at 4 °C, and the H_2_O_2_ content in the supernatant was determined using an H_2_O_2_-2-Y kit (Suzhou Comin) following the manufacturer’s instructions.

### 2.6. Heterologous Biosynthesis of S. baumii Triterpenoid Under Ca^2+^ Induction

To investigate the effect of Ca^2+^ on triterpenoid biosynthesis in yeast, yeast cells harboring the empty pYES2-NTC vector were treated with various concentrations of Ca^2+^ to determine the optimal Ca^2+^ concentration for triterpenoid synthesis. Yeast harboring the empty pYES2-NTC vector served as the control (Ck1) group. Pearson correlation analysis was performed to assess the correlation between triterpenoid content and the expression levels of triterpenoid synthesis genes in *S. baumii* on the basis of transcriptomic data. The key gene involved in triterpenoid biosynthesis was selected and cloned (Table S1). The cloned gene was ligated into the pYES2-NTC vector using homologous recombination to create a new vector, pYES2-AACT. This vector was then transformed into competent yeast cells to obtain the new strain Sc-AA1.

Strain Sc-AA1 was cultivated and treated with the Ca^2+^ concentration optimal for triterpenoid production; the thallus was harvested via centrifugation and used to determine the content of squalene. A pretreatment step was required to facilitate the extraction of triterpenoids from yeast. In brief, 0.1 g of thallus was placed into a 10 mL centrifuge tube, and 2 mL of a 10% KOH–75% ethanol solution was added; the tube was then boiled for 15 min. Subsequently, 2 mL of *n*-hexane was added to the centrifuge tube and mixed well, and the mixture was allowed to stratify. The supernatant was transferred, and the extraction process was repeated with another 2 mL of *n*-hexane. All supernatants were combined and evaporated at 60 °C. Then, 1 mL of acetonitrile was added, and the solution was used for UHPLC analysis [22,23]. UHPLC analysis was performed on an Agilent 1290 system equipped with a C18 column (5C18-PAQ, 4.6 × 250 mm, COSMOSIL, Tokyo, Japan). The column temperature was maintained at 20 °C, and the mobile phase consisted of 100% acetonitrile at a flow rate of 1 mL/min under isocratic elution. Detection was carried out using a diode array detector at 203 nm. The squalene content was calculated from a standard curve (y = 3.4133x + 14.731, R^2^ = 0.9999) using squalene.

### 2.7. Statistics and Analysis

Experimental data, including mycelial growth rate, Ca^2+^ content, triterpenoid content, gene expression, and metabolic content, were derived from three biological replicates. The significance of differences between samples was tested using Duncan’s test and SPSS software v17.0 (SPSS Inc., Chicago, IL, USA). Differences were considered significant at *p* < 0.05, and data are presented as mean ± standard deviation (SD). Pearson correlation analysis was performed to calculate the correlation coefficient between gene expression levels and triterpenoid/Ca^2+^ content; R > 0 indicates a positive correlation, and R < 0 indicates a negative correlation.

## 3. Results

### 3.1. Effect of Ca^2+^ Treatment on Mycelial Growth and Triterpenoid Content in S. baumii

**Figure 1:**
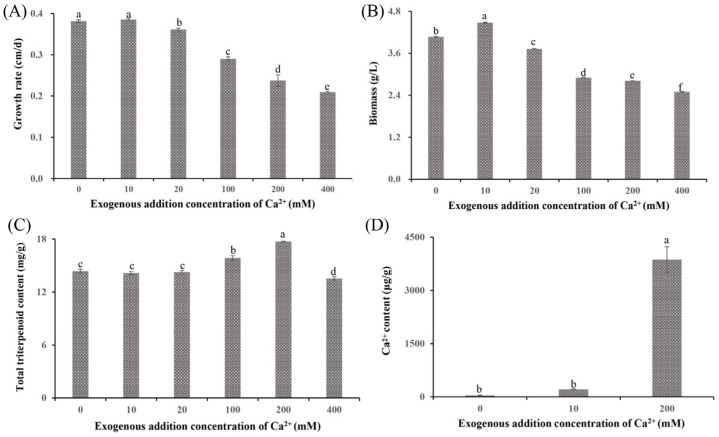
Growth characteristics and total triterpenoid content of *Sanghuangporus baumii* mycelia treated with various concentrations of Ca^2+^. (**A**) Mycelial growth rate; (**B**) biomass; (**C**) total triterpenoid content in *S. baumii* mycelia; (**D**) Ca^2+^ content in *S. baumii* mycelia. Different letters share significant difference, *p* < 0.05.

Previous studies have reported that Ca^2+^ affects both growth and metabolite synthesis [13,24]. In this study, significant phenotypic differences were observed in *S. baumii* mycelia treated with various Ca^2+^ concentrations (Figure 1A and Figure S1). The 10 mM Ca^2+^ (the Ca10 group) promoted mycelial growth at a rate of 0.39 cm/d, which was faster than that in the 0 mM Ca^2+^ treatment (Ca0 group). However, at Ca^2+^ concentrations >10 mM, the growth rates decreased in a concentration-dependent manner, with the Ca400 group showing a growth rate of only 0.21 cm/d. Biomass exhibited a similar trend, with the Ca10 group showing the highest biomass at 4.48 g/L and the Ca400 group showing the lowest biomass at 2.50 g/L (Figure 1B). Notably, treatment with 10 mM Ca^2+^ did not alter the triterpenoid content (14.15 mg/g); in contrast, the Ca200 group showed a significantly higher triterpenoid content (17.71 mg/g) than the Ca0 group (Figure 1C). Analysis of the Ca^2+^ content indicated that Ca^2+^ was absorbed by *S. baumii* mycelia (Ca200 group, 3869.97 µg/g; Figure 1D), suggesting the involvement of Ca^2+^ in both mycelial growth and changes in triterpenoid content.

### 3.2. Transcriptome Analysis of Metabolic Pathways and Differential Gene Expression in S. baumii Mycelia Treated with Ca^2+^

**Figure 2:**
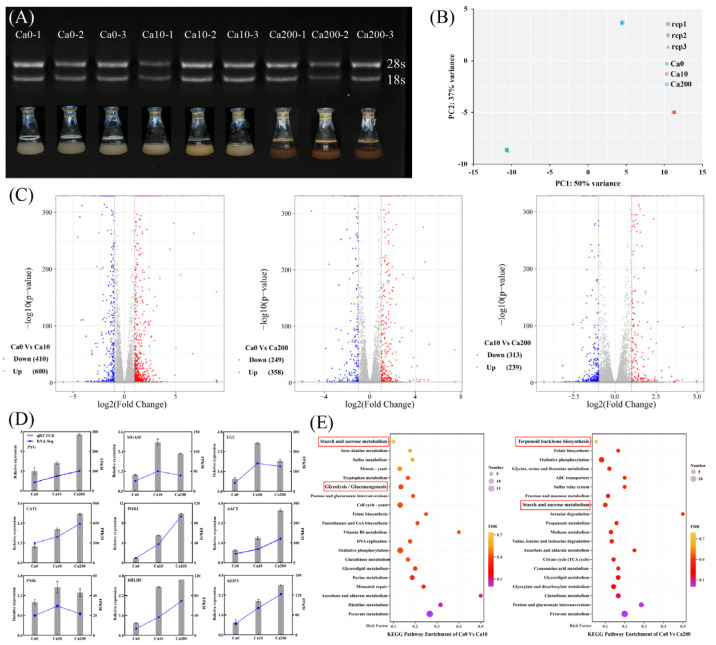
Transcriptome analysis to analyze the differences in growth characteristics and metabolite synthesis between *Sanghuangporus baumii* mycelia treated with various Ca^2+^ concentrations. (**A**) RNA gel electrophoresis of nine samples (three replicates each of the 0 mM Ca^2+^ [Ca0], 10 mM Ca^2+^ [Ca10], and 200 mM Ca^2+^ treatment groups [Ca200]); (**B**) PCA score plot of transcript profiles of the Ca0, Ca10, and Ca200 groups; (**C**) volcano plot of upregulated and downregulated genes identified in pairwise comparisons; (**D**) qRT-RCR validation of gene expression; (**E**) KEGG enrichment analysis of DEGs from pairwise comparisons.

To elucidate the mechanism by which Ca^2+^ affects the growth and triterpenoid biosynthesis of *S. baumii*, transcriptome analysis was performed using high-quality RNA extracted from *S. baumii* (Figure 2A). The sequencing data exhibited adequate saturation (Figure S2), with a Q20 value >98.62% and a Q30 value >95.96% (Table S2). PCA confirmed the high reproducibility of the data (Figure 2B); thus, the data were considered suitable for subsequent differential expression analysis. The analysis identified a total of 1010 DEGs (600 upregulated and 410 downregulated) between the Ca0 and Ca10 groups, 607 DEGs (358 upregulated and 249 downregulated) between the Ca0 and Ca200 groups, and 552 DEGs (239 upregulated and 313 downregulated) between the Ca10 and Ca200 groups (Figure 2C). The relative expression profiles of DEGs determined by qRT-PCR were consistent with the corresponding FPKM values derived from the transcriptomic data (Figure 2D; Table S1). KEGG enrichment analysis demonstrated that the DEGs between the Ca0 and Ca10 groups were enriched in the starch and sucrose metabolism pathway and the glycolysis/gluconeogenesis pathway, whereas the DEGs between the Ca0 and Ca200 groups were enriched in the starch and sucrose metabolism pathway and the terpenoid backbone biosynthesis pathway. These results suggest that the differences in growth and metabolite synthesis in *S. baumii* mycelia are closely associated with these metabolic pathways and the DEGs associated with them.

### 3.3. Analysis of Growth Differences in S. baumii Mycelia Under Ca^2+^ Treatment

**Figure 3:**
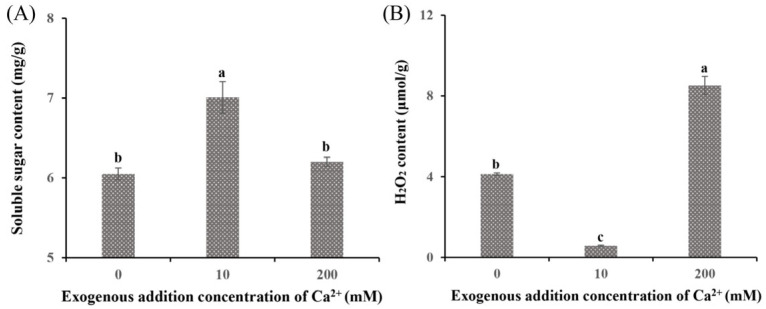
Soluble sugar and H_2_O_2_ content of *Sanghuangporus baumii* treated with various Ca^2+^ concentrations. (**A**) Soluble sugar content; (**B**) H_2_O_2_ content. Different letters share significant difference, *p* < 0.05.

The DEGs between the Ca0 group and the Ca10 treatment group were enriched in the starch and sucrose metabolism pathway and the glycolysis/gluconeogenesis pathway. The soluble sugar content (7.01 mg/g; Figure 3A) and the expression levels of related genes (*PYG*, *MGAM*, and *EG*2; Table S3) in the Ca10 group were markedly higher than those in the Ca0 group, indicating their critical role in enhancing the growth rate and biomass of *S. baumii* mycelia. In contrast, the addition of 200 mM Ca^2+^ inhibited mycelial growth, and the Ca200 group showed a significantly higher H_2_O_2_ content (8.52 µmol/g; Figure 3B) and higher expression levels of associated genes (*CAT1* and *POD1*; Table S3) than the Ca0 group. This finding suggests that excessive H_2_O_2_ production is detrimental to *S. baumii* mycelial growth.

### 3.4. Mechanism of Ca^2+^-Induced Increase in the Triterpenoid Content of S. baumii

**Figure 4:**
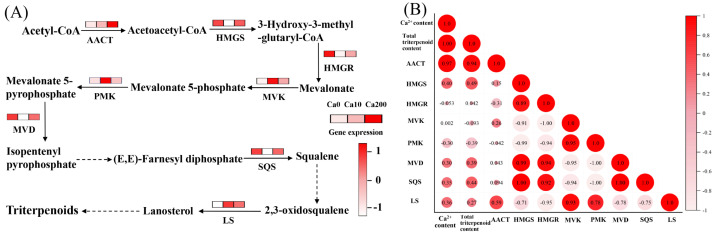
Ca^2+^-induced biosynthesis of triterpenoids in *Sanghuangporus baumii*. acetyl-CoA acyltransferase, AACT; 3-hydroxy-3-methylglutaryl-CoA synthase, HMGS; 3-hydroxy-3-methylglutaryl-CoA reductase, HMGR; mevalonate kinase, MVK; phosphomevalonate kinase, PMK; mevalonate pyrophosphate decarboxylase, MVD; squalene synthase, SQS; lanosterol synthase, LS. (**A**) Expression levels of genes associated with triterpenoid biosynthesis; (**B**) results of Pearson correlation analysis among Ca^2+^ content, total triterpenoid content, and expression levels of key genes involved in triterpenoid biosynthesis.

Treatment with 10 mM Ca^2+^ did not significantly affect triterpenoid synthesis in *S. baumii*. However, treatment with 200 mM Ca^2+^ significantly increased the total triterpenoid content, primarily by activating the terpenoid biosynthesis pathway and enhancing the expression of related genes (Figure 4A). Pearson correlation analysis showed a significant positive correlation between Ca^2+^ concentration and total triterpenoid accumulation (correlation coefficient 1.0; Figure 4B), indicating that Ca^2+^ plays a role in regulating triterpenoid biosynthesis in *S. baumii*. Further analysis identified the key genes regulating triterpenoid biosynthesis; the results highlighted that total triterpenoid accumulation was most strongly correlated with *AACT* gene expression (correlation coefficient 0.94) (Figure 4B). This suggests that the *AACT* gene is actively involved in triterpenoid biosynthesis under Ca^2+^ regulation.

### 3.5. Preliminary Realization of Heterologous Biosynthesis of S. baumii Triterpenoid in Yeast

**Figure 5:**
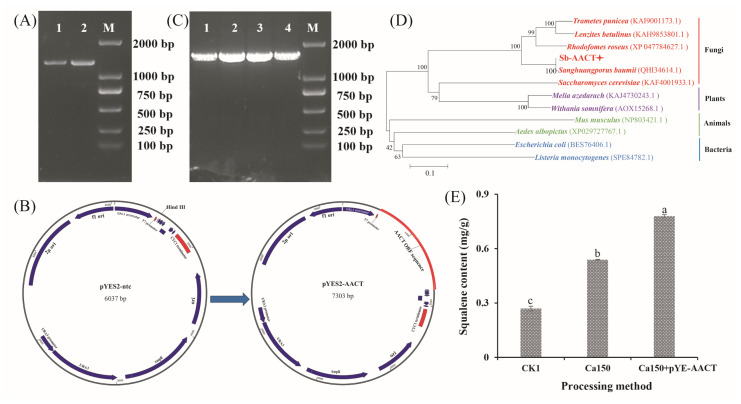
Heterologous biosynthesis of *Sanghuangporus baumii* triterpenoid in yeast. (**A**) *AACT* gene cloning; (**B**) construction of the *AACT* gene overexpression vector; (**C**) detection of positive transformants; (**D**) homologous alignment analysis of the *AACT* gene; (**E**) squalene content in yeast (Ck1, yeast cells transformed with pYES2-NTC; Ca150, cells transformed with pYES2-NTC treated with 150 mM Ca^2+^; Ca150+pYE-AACT, cells expressing the *AACT* gene and treated with 150 mM Ca^2+^). Different letters share significant difference, *p* < 0.05.

Ca^2+^ treatment not only affected the triterpenoid biosynthesis pathway in *S. baumii* but also facilitated the accumulation of *S. baumii* triterpenoids in yeast. Notably, treatment with 150 mM Ca^2+^ significantly elevated the squalene content in yeast, reaching 0.54 mg/g (Figure S3). To further increase the squalene content, the *AACT* gene, identified as pivotal for triterpenoid biosynthesis in *S. baumii*, was cloned (Figure 5A) and inserted into pYES2-NTC to create the vector pYES2-AACT (Figure 5B), which was subsequently transformed into competent yeast cells. Positive transformants were confirmed through PCR detection, yielding a product of approximately 1600 base pairs (Figure 5C). Sequencing results confirmed the successful integration of the *AACT* gene into yeast (Figure 5D), confirming the development of the novel strain Sc-AA1. Strain Sc-AA1 cultured with 150 mM Ca^2+^ yielded a squalene content of 0.78 mg/g (Figure 5E), which is 2.89-fold higher than that in the control (Ck1) group. These findings indicate that the heterologous biosynthesis of *S. baumii* triterpenoids in yeast was successfully achieved.

## 4. Discussion

Ca^2+^ has been reported to regulate fungal growth, with low concentrations promoting mycelial development [25]. Consistent with previous studies, the present study demonstrated that the growth rate and biomass of *S. baumii* mycelia treated with 10 mM Ca^2+^ were higher than those in the Ca0 group (by 1.03% and 10.05%, respectively) (Figure 1A,B). This enhancement in the Ca10 group may be attributed to a significant increase in soluble sugar content, which provides energy for mycelial growth [26]. In contrast, treatment with 200 mM Ca^2+^ decreased the mycelial growth rate and biomass, likely because of H_2_O_2_ accumulation, which resulted in higher toxicity within *S. baumii* mycelia (Figure 3B). Such reactive oxygen species are considered toxic cellular waste [27].

Ca^2+^ has been reported to not only regulate growth but also influence the production of secondary metabolites [24]. A previous study showed that Ca^2+^ treatment of *G. lucidum* significantly enhanced the expression of the *sqs* gene and increased the triterpenoid content by 3.7-fold [13]. Consistent with this finding, in the present study, Ca^2+^ treatment resulted in a 1.23-fold increase in total triterpenoid content in *S. baumii* mycelia, with the *AACT* gene—rather than the *sqs* gene—showing a significant positive correlation with the changes in total triterpenoid content. Therefore, we investigated the effects of Ca^2+^ and the *S. baumii AACT* gene on triterpenoid synthesis in yeast. The results indicated that 150 mM Ca^2+^ treatment increased the squalene content by 2-fold, and in yeast harboring the *AACT* gene, the squalene content was 2.89-fold higher than that in the Ck1 group (Figure 5E). These findings are important for the preliminary realization of the heterologous biosynthesis of *S. baumii* triterpenoid. Currently, the heterologous biosynthesis of *S. baumii* triterpenoid does not meet the requirements for large-scale production, potentially because of insufficient expression levels of individual genes [16,17,28]. Heterologous biosynthesis has emerged as a promising approach in metabolite synthesis, and using multigene transfer approaches may further enhance the yield.

## 5. Conclusions

Our study first confirmed that 10 mM Ca^2+^ positively affects the growth rate and biomass accumulation of *S. baumii* mycelia. This effect is primarily due to the stimulation of soluble sugar accumulation and the expression of related genes, which provide sufficient energy for the growth of *S. baumii* mycelia. Conversely, 200 mM Ca^2+^ is detrimental to growth and biomass accumulation due to a modest increase in H_2_O_2_ content, which induces toxicity. However, 200 mM Ca^2+^ also offers certain advantages, as it can promote the accumulation of triterpenoid in both *S. baumii* and yeast. The optimal combination of Ca^2+^ concentration and overexpression of the *AACT* gene can further enhance squalene content. The preliminary research results demonstrate successful heterologous biosynthesis of *S. baumii* triterpenoid, which provides important guidance for the long-term stable production of these compounds.

## Data Availability

The original contributions presented in this study are included in the article/Supplementary Materials. Further inquiries can be directed to the corresponding author.

## Supplementary Materials

The following supporting information can be downloaded at https://www.mdpi.com/article/10.3390/jof11030238/s1, Figure S1: Colony morphology of *S. baumii* treated with various concentrations of Ca^2+^; Figure S2: Sequencing saturation of nine samples; Figure S3: Squalene content in yeast that contains the pYES2-NTC vector treated with different Ca^2+^ concentrations. Different letters share significant difference, *p* < 0.05; Table S1: Information about primers used in the present study; Table S2: RNA sequencing data statistics for *S. baumii*; Table S3: Transcript abundance of related genes according to the *S. baumii* transcriptome data annotation.
