# Supplementary material for: Calcium-Induced Regulation of Sanghuangporus baumii Growth and the Biosynthesis of Its Triterpenoids
Source: J Fungi (Basel). 2025 Mar 20;11(3):238. doi: 10.3390/jof11030238 (PMC11943593; doi:10.3390/jof11030238)
Supplement: Supplementary file 1 [file jof-3518985-supplementary.pdf › jof-3518985-supplementary.pdf]

Supplementary Material

1. Supplementary Figure

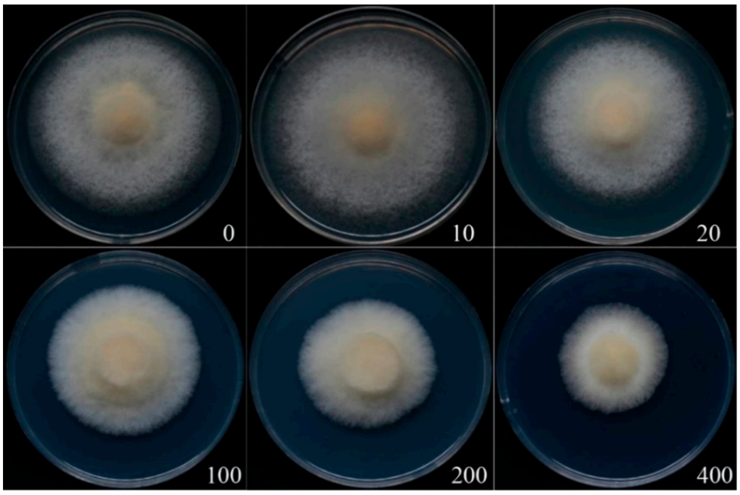

Figure S1. Colony morphology of *S. baumii* treated with various concentrations of Ca^{2+}.

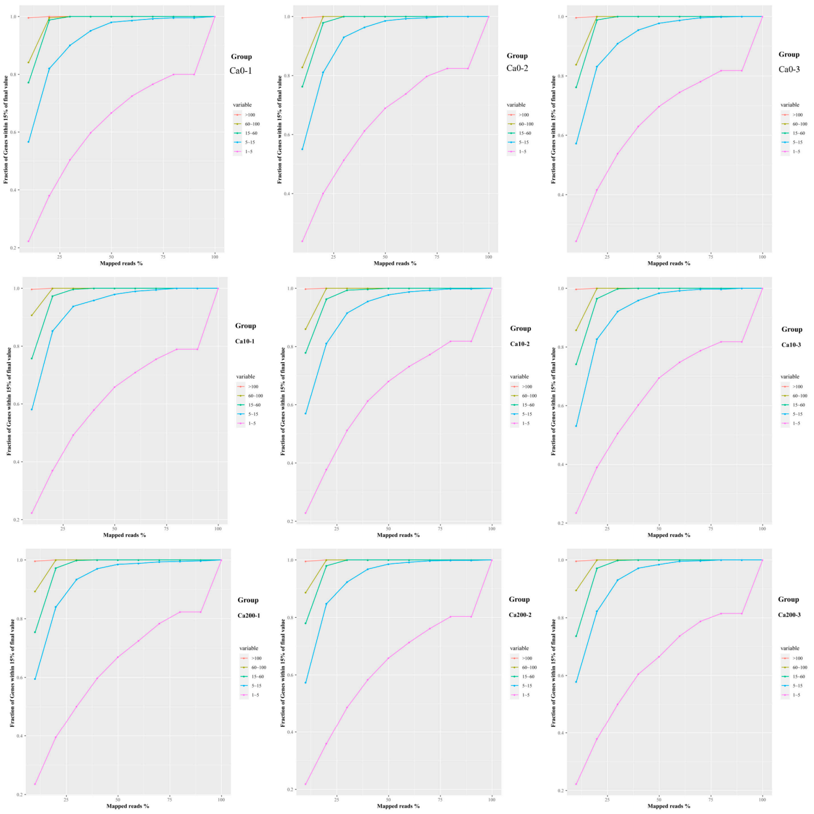

Figure S2. Sequencing saturation of nine samples.

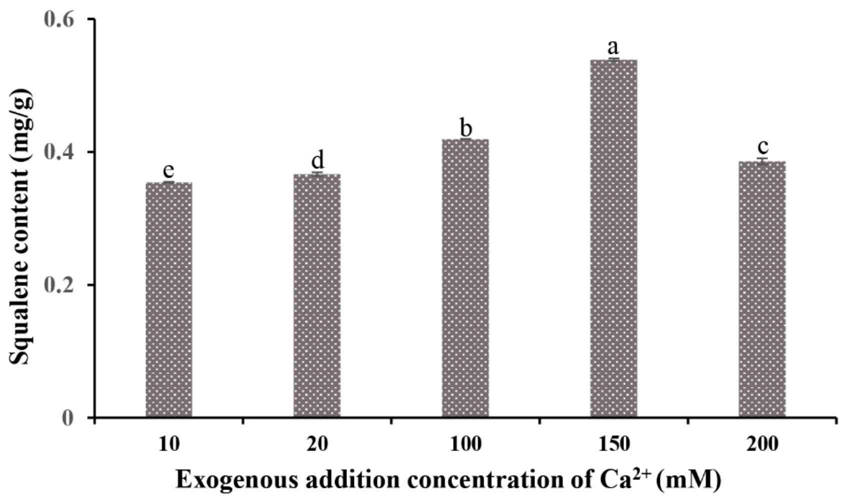

Figure S3. Squalene content in yeast containing the pYES2-NTC vector treated with different Ca^{2+} concentrations. Different letters share significant difference, $p < 0.05$.

2. Supplementary Table

Table S1 Information about primers used in the present study.

Primers	Sequences 5′→3′	Size (bp)	Annotation
PYG-F	CGGTGTCGTTCATCGTTATTT	137	qRT-PCR
PYG-R	CGATCAAGCGGATGGTCAG		
MGAM-F	TGGTGGACGGAGGCATTTAGGA	148	qRT-PCR
MGAM-R	GGATGAACGGCGTGGAAGTATT		
EG2-F	TCTCTATGGTCTCAAGTCGCCC	124	qRT-PCR
EG2-R	TTACTGCTGCTTGGACAGAACC		
CAT1-F	ACCTGTCAACGCACCCATTGTA	138	qRT-PCR
CAT1-R	GCATCGTGGTAGGGCTTCTTCT		
POD1-F	GGCATCGCCTTCTCTCCAACTC	120	qRT-PCR
POD1-R	AACGATGTCCTCAACACCGCCG		
AACT-F	TCGGTGTCAAACCACTCGCAAAAAT	285	qRT-PCR
AACT-R	CCAGAGAAACGATAATACGGCAACC		
PMK-F	AAGATTGAGCCATTCCAACTCCCAC	113	qRT-PCR
PMK-R	GCAGCCTTATGCCACTTCAAAACTT		
bHLH5-F	ACAGAAGACCATTGACTACATCCAC	166	qRT-PCR
bHLH5-R	AGTGATACCCGTCCCACCAGTC		
bZIP3-F	GGATCAGAATCGAATCGCCCAAC	95	qRT-PCR
bZIP3-R	TGCTGCCAGACAGAATTTCAACG		
α-tubulin-F	CCAGCAAGCGTTACCGATT	129	qRT-PCR
α-tubulin-R	TCCACGACGTCCATCGTTC		
pYE-AACT-F	AGGGAATATTAAGCTTATGTTCGCCGCCCGTTCC	1292	gene clone
pYE-AACT-R	CCCCCATGGTAAGCTTTCACAGCTTCTGGATCACAAGAGC		
T7F	TAATACGACTCACTATAGGG	329	detection of transformants
C1R	GTGACATAACTAATTACATGATG		

Table S2 RNA sequencing data statistics for *S. baumii*.

Sample	Reads No.	Q20(%)	Q30(%)	Clean Reads No.	Clean Reads %
Ca0-1	42642556	98.78	96.39	42122170	98.78
Ca0-2	43600618	98.66	96.07	42967442	98.55
Ca0-3	45811882	98.78	96.37	45263134	98.8
Ca10-1	44352516	98.78	96.38	43805920	98.77
Ca10-2	45514104	98.7	96.18	44877504	98.6
Ca10-3	53976642	98.79	96.39	53334052	98.81
Ca200-1	44945184	98.75	96.3	44303818	98.57
Ca200-2	37096474	98.67	96.06	36604754	98.67
Ca200-3	41176080	98.62	95.96	40576132	98.54

Table S3 Transcript abundance of related genes as per the *S. baumii* transcriptome data annotation

Pathway	Gene ID	Gene name	EC.No.	Ca0-1	Ca0-2	Ca0-3	Ca10-1	Ca10-2	Ca10-3	Ca200-1	Ca200-2	Ca200-3
Starch and sucrose metabolism	OCB85998.1	PYG	2.4.1.1	43.14	42.71	43.58	75.27	74.62	74.84	100.29	99.59	102.56
	OCB89527.1	MGAM	3.2.1.20	25.85	25.56	26.86	51.89	50.44	49.49	41.01	38.28	40.18
	OCB90205.1	EG2	3.2.1.4	42.12	41.29	43.25	134.77	142.8	145.9	126.93	126.22	122.86
Peroxisome	OCB88914.1	SOD2	1.15.1.1	10.8	13.6	12.38	36.21	34.81	40.15	18.79	18.8	16.35
	OCB92026.1	CAT1	1.11.1.6	294.78	295.51	300.78	395.81	390.79	390.81	582.6	586.43	597.22
	OCB89183.1	POD1	-	9.6	9.71	9.47	38.51	37.82	36.31	91.99	93.54	95.43
Terpenoid backbone biosynthesis	QHI34614.1	AACT	2.3.1.9	43.79	45.39	44.63	67.51	67.22	68.68	129.33	118.31	114.21
	QIJ32333.1	PMK	2.7.4.2	20.95	19.69	18.87	29.04	28.21	31.13	20.98	23.39	20.61
